# Neuroprotective and Anti-Inflammatory Effects of *Pinus densiflora* Bark Extract in Gerbil Hippocampus Following Transient Forebrain Ischemia

**DOI:** 10.3390/molecules26154592

**Published:** 2021-07-29

**Authors:** Joon Ha Park, Jong Dai Kim, Tae-Kyeong Lee, Xionggao Han, Hyejin Sim, Bora Kim, Jae-Chul Lee, Ji Hyeon Ahn, Choong-Hyun Lee, Dae Won Kim, Moo-Ho Won, Soo Young Choi

**Affiliations:** 1 Department of Anatomy, College of Korean Medicine, Dongguk University, Gyeongju 38066, Korea; jh-park@dongguk.ac.kr; 2 Division of Food Biotechnology, School of Biotechnology, Kangwon National University, Chuncheon 24341, Korea; jongdai@kangwon.ac.kr (J.D.K.); paran312@hanmail.net (X.H.); 3 Department of Biomedical Science and Research Institute for Bioscience and Biotechnology, Hallym University, Chuncheon 24252, Korea; tk_lee@hallym.ac.kr; 4 Department of Neurobiology, School of Medicine, Kangwon National University, Chuncheon 24341, Korea; janny20@naver.com (H.S.); nbrkim17@gmail.com (B.K.); anajclee@kangwon.ac.kr (J.-C.L.); jh-ahn@ysu.ac.kr (J.H.A.); 5 Department of Physical Therapy, College of Health Science, Youngsan University, Yangsan 50510, Korea; 6 Department of Pharmacy, College of Pharmacy, Dankook University, Cheonan 31116, Korea; anaphy@dankook.ac.kr; 7 Department of Biochemistry and Molecular Biology, Research Institute of Oral Sciences, College of Dentistry, Gangnung-Wonju National University, Gangneung 25457, Korea; kimdw@gwnu.ac.kr

**Keywords:** flavonoids, hippocampus, inflammatory cytokines, microgliosis, polyphenols, proanthocyanidins, pyramidal neuron

## Abstract

Korean red pine (*Pinus densiflora*) belongs to the Genus *Pinus,* and its bark contains a great amount of naturally occurring phenolic compounds. Until now, few studies have been conducted to assess the neuroprotective effects of *Pinus densiflora* bark extract against brain ischemic injury. The aim of this study was to investigate the neuroprotective effects of pre-treatment with the extract in the hippocampus following 5-min transient forebrain ischemia in gerbils. Furthermore, this study examined the anti-inflammatory effect as a neuroprotective mechanism of the extract. *Pinus densiflora* bark was extracted by pure water (100 °C), and this extract was quantitatively analyzed and contained abundant polyphenols, flavonoids, and proanthocyanidins. The extract (25, 50, and 100 mg/kg) was orally administered once a day for seven days before the ischemia. In the gerbil hippocampus, death of the pyramidal neurons was found in the subfield cornu ammonis 1 (CA1) five days after the ischemia. This death was significantly attenuated by pre-treatment with 100 mg/kg, not 25 or 50 mg/kg, of the extract. The treatment with 100 mg/kg of the extract markedly inhibited the activation of microglia (microgliosis) and significantly decreased the expression of pro-inflammatory cytokines (interleukin 1β and tumor necrosis factor α). In addition, the treatment significantly increased anti-inflammatory cytokines (interleukin 4 and interleukin 13). Taken together, this study clearly indicates that pre-treatment with 100 mg/kg of *Pinus densiflora* bark extract in gerbils can exert neuroprotection against brain ischemic injury by the attenuation of neuroinflammatory responses.

## 1. Introduction

Transient ischemia in brains occurs when blood supply to the brain is temporarily lost [1]. The most effective treatment for ischemic brains is to restore blood supply to the brains as soon as possible. However, when the restoration of blood supply is too late, ischemia-reperfusion injury leads to delayed neuronal death in vulnerable brain structures, such as the hippocampal CA1 [2,3]. Numerous studies have proposed diverse mechanisms of the delayed neuronal death due to ischemia-reperfusion injury, including glutamate-induced excitotoxicity, oxidative stress following excessive generation of reactive oxygen species (ROS), glia-mediated neuroinflammation, and blood-brain barrier (BBB) disruption [4,5,6]. However, the exact mechanism of the delayed neuronal death has not been fully elucidated.

Plant extracts have received considerable attention as the potential sources of multi-target therapeutic agents for the treatment of neurological diseases, including cerebral ischemia because they have multiple health-beneficial components that display a wide spectrum of biological properties [7,8]. Pine bark extract has been studied to have usable polyphenols, including proanthocyanidins, which display various pharmacological properties [9,10]. Thus, pine bark extract has been traditionally used for various diseases and is utilized for nutritional supplements [11,12].

Several reports have demonstrated that pine trees inhabiting coastal regions display various beneficial properties [13,14]. For a representative example, Pycnogenol^®^, a standardized extract from French maritime pine bark (*Pinus pinaster* Aiton), exerts a neuroprotective effect via antioxidant efficacy in a gerbil model of transient forebrain ischemia [13]. However, to the best of our knowledge, there is little information available on the neuroprotective effect of an extract from Korean red pine (*Pinus densiflora*) bark against ischemic brain injury. In addition, the mechanisms underlying the neuroprotective effects of the extract against ischemic injury have not yet been fully established. Therefore, the present study aimed to investigate the neuroprotective effect of Korean red pine bark extract in the hippocampus and whether its effect occurred via the anti-inflammatory function of the extract following 5-min transient forebrain ischemia in gerbils that have been used as a model for evaluating the efficacy and mechanisms of protective agents in transient forebrain ischemia [15,16].

## 2. Results

### 2.1. Total Polyphenols, Flavonoids, and Proanthocyanidins of Pinus densiflora Bark Extract (PBE)

As shown in Table 1, total polyphenols, total flavonoids, and total proanthocyanidins contained in PBE were 92.89 ± 066 mg GAE/g, 23.57 ± 0.11 mg QE/g, and 53.42 ± 6.47 mg CE/g, respectively (Table 1).

### 2.2. PBE Protected Hippocampal Neurons from Ischemic Injury

#### 2.2.1. Finding by Hematoxylin and Eosin (H&E) Staining

**Figure 1:**
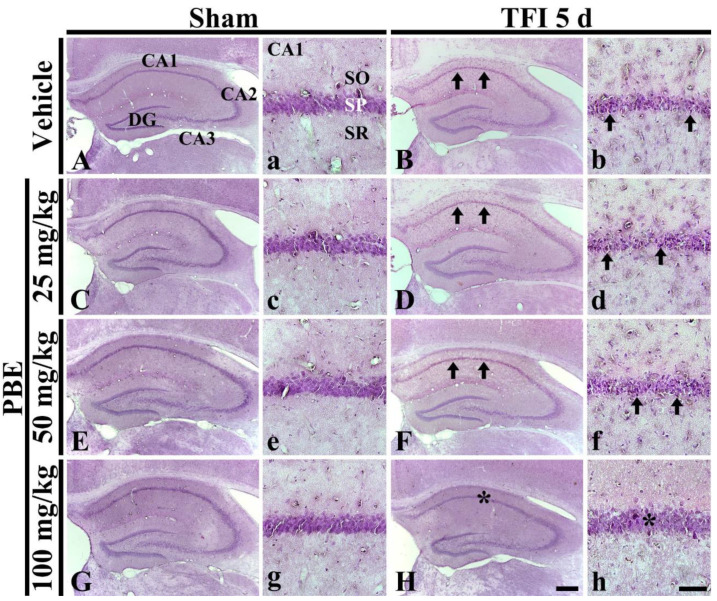
Representative images of H&E staining in gerbil hippocampus obtained from the vehicle + sham (**A**,**a**), vehicle + TFI (**B**,**b**), PBE (25 mg/kg) + sham (**C**,**c**), PBE (25 mg/kg) + TFI (**D**,**d**), PBE (50 mg/kg) + sham (**E**,**e**), PBE (50 mg/kg) + TFI (**F**,**f**), PBE (100 mg/kg) + sham (**G**,**g**), and PBE (100 mg/kg) + TFI (**H**,**h**) groups at 5 days post-ischemia. Cells located in the SP (arrows in (**B**,**b**,**D**,**d**,**F**,**f**)) in the vehicle + TFI and PBE (25 and 50 mg/kg) + TFI groups are apparently damaged. However, in the PBE (100 mg/kg) + TFI group, H&E stainability (asterisks in **H**,**h**) in the SP is similar to that found in the vehicle + sham group. DG, dentate gyrus; SO, stratum oriens; SR, stratum radiatum. Scale bar = 400 μm (**A**)–(**H**), 100 μm (**a**)–(**h**).

In the vehicle-treated and sham-operated (vehicle + sham) group, cells in the hippocampus were well-stained with H&E, showing that the cells basically consisted of the stratum pyramidale (SP) in CA1-3 (Figure 1a). In the vehicle-treated and transient forebrain ischemia (TFI) operated (vehicle + TFI) group, cells stained with H&E in the SP of CA1, not CA2/3, were smaller in size, and there was a lack of cytoplasm at 5 days post-ischemia compared to those shown in the vehicle + sham group (Figure 1B,b). This finding means that the cells of the CA1 SP (called CA1 pyramidal cells or neurons) are damaged or dead.

In the PBE (25, 50, and 100 mg/kg)-treated and sham-operated (PBE + sham) groups, H&E stainability in the hippocampus was similar to that shown in the vehicle + sham group at 5 days post-ischemia (Figure 1C,c,E,e,G,g). In the PBE (25 and 50 mg/kg) + TFI groups, H&E stainability in the hippocampus was similar to that shown in the vehicle + TFI group (Figure 1D,d,F,f). However, in the PBE (100 mg/kg) + TFI group, H&E stainability was similar to that found in the vehicle + sham group (Figure 1H,h). Based on these results, pre-treatment with 100 mg/kg PBE showed a neuroprotective effect in gerbil hippocampal CA1 following TFI, and therefore, its underlying mechanisms were investigated in the PBE (100 mg/kg) + TFI group.

#### 2.2.2. Finding by Neuron-Specific Soluble Nuclear Antigen (NeuN) Immunohistofluorescence

**Figure 2:**
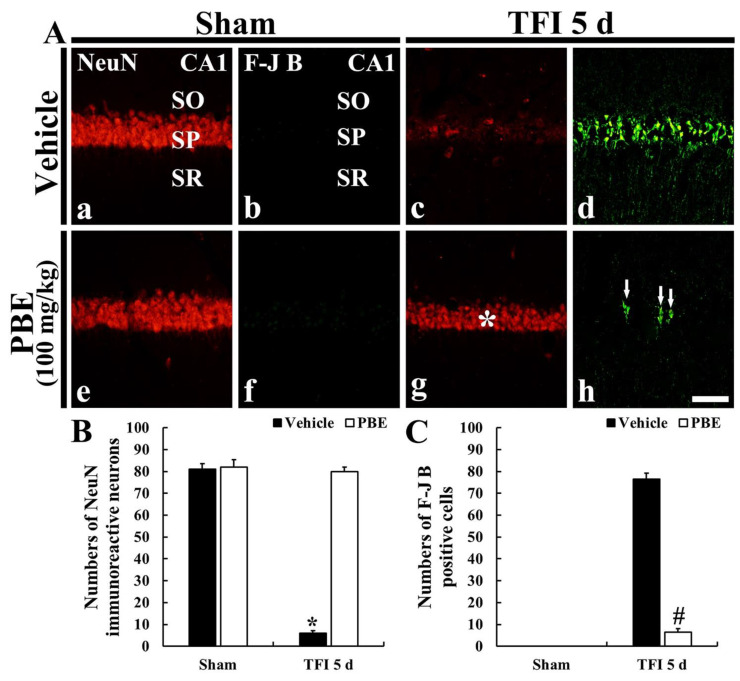
(**A**) Representative images of NeuN immunofluorescence (**a**,**c**,**e**,**g**) and F-J B histofluorescence (**b**,**d**,**f**,**h**) staining in CA1 of the vehicle + sham (**a**,**b**), vehicle + TFI (**c**,**d**), PBE + sham (**e**,**f**), and PBE + TFI (**g**,**h**) groups at 5 days TFI. In the vehicle + TFI group, a few NeuN^+^ and many F-J B^+^ cells are shown in the SP. However, in the PBE + TFI group, numerous NeuN^+^ cells (asterisk in (**g**)) and a few F-J B^+^ cells (arrows in (**h**)) are shown in the SP. Scale bar = 100 μm. (**B**,**C**) Mean numbers of NeuN^+^ (**B**) and F-J B^+^ (**C**) cells in the SP at 5 days post-ischemia. The bars indicate the means ± SEM (*n* = 7/group; * *p* < 0.05 versus each sham group, **^#^** *p* < 0.05 versus vehicle + TFI group).

In the vehicle + sham and PBE + sham groups, CA1 pyramidal cells located in the SP showed strong NeuN immunofluorescence, which is shown in intact neurons (Figure 2Aa,Ae. In the vehicle + TFI group, NeuN immunoreactive (NeuN^+^) CA1 pyramidal cells were rarely shown (about 6 cells/250 μm^2^) at 5 days post-ischemia (Figure 2Ac,B. However, in the PBE + TFI group, NeuN^+^ CA1 pyramidal neurons were similar to those found in the vehicle + sham group (Figure 2Ag,B).

#### 2.2.3. Finding by Fluoro-Jade B (F-J B) Histofluorescence

F-J B positive (F-J B^+^) cells, which are dead or degenerating cells, were not detected in the vehicle + sham and PBE + sham groups (Figure 2Ab,Af). In the vehicle + TFI group, many F-J B^+^ cells (about 77 cells/250 μm^2^) were observed in the SP at 5 days post-ischemia (Figure 2Ad,C). However, in the PBE + TFI group, a few F-J B^+^ cells (about 7 cells/250 μm^2^) were detected in the SP at 5 days after TFI (Figure 2Ah,C).

### 2.3. PBE Attenuated Microglia Activation (Microgliosis) in Ischemic CA1

**Figure 3:**
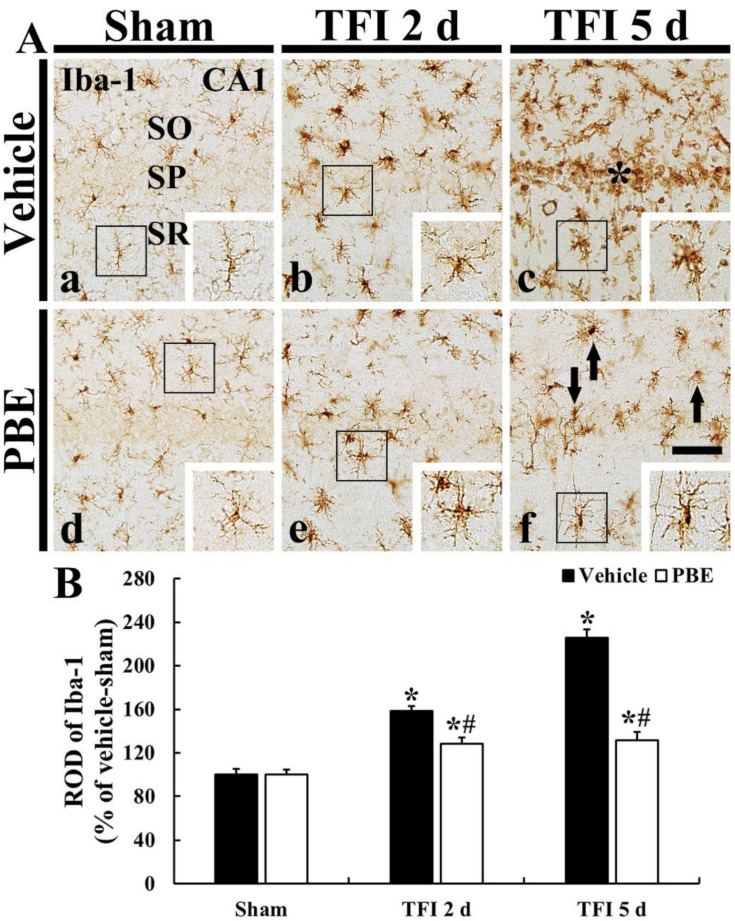
(**A**) Representative images of Iba-1 immunohistochemistry in CA1 of the vehicle + sham (**a**), vehicle + TFI (**b**,**c**), PBE + sham (**d**), and PBE + TFI (**e**,**f**) groups at 2 days and 5 days post-ischemia. In the vehicle + TFI group, Iba-1+ microglia are hypertrophied after TFI. Note that hypertrophied Iba-1+ microglia are clustered in the SP at 5 days post-ischemia. However, in the PBE + TFI group, the activation of Iba-1+ microglia are significantly attenuated (arrows) (about 58% of that shown in the vehicle + TFI group) at 5 days post-ischemia. Scale bar = 100 μm. (B) ROD of Iba-1+ structures. The bars indicate the means ± SEM (*n* = 7 at each time; * *p* < 0.05, vs. vehicle + sham group, ^#^ *p* < 0.05 vs. corresponding vehicle + TFI group).

In the vehicle + sham group, ionized calcium-binding adapter molecule 1 (Iba-1) immunoreactive (Iba-1^+^) microglia were scattered in strata oriens (SO) and radiatum (SR) (Figure 3Aa). They were typical in morphology (as a resting form, small cell body with long branched processes) (Figure 3Aa). In the vehicle + TFI group, Iba-1^+^ microglia were activated (hypertrophied: enlarged cell bodies with shorter and thicker processes), and the relative optical density (ROD) of Iba-1^+^ microglia was gradually and significantly increased (158.4% at 2 days and 225.5% at 5 days after TFI) compared to that in the vehicle + sham group (Figure 3A(b,c),B). In particular, at 5 days post-ischemia, many activated Iba-1^+^ microglia were clustered in the SP (Figure 3Ac).

In the PBE + sham group, Iba-1^+^ microglia were not different in their morphology and distribution from that shown in the vehicle + sham group (Figure 3Ad). In the PBE + TFI group, Iba-1^+^ microglia activation was significantly attenuated when compared with that found in the vehicle + TFI group at 5 days post-ischemia, and the ROD was 80.8% at 2 days and 58.4% at 5 days compared with that shown in the vehicle + TFI group (Figure 3A(e,f),B).

### 2.4. PBE Decreased Pro-Inflammatory Cytokines in Ischemic CA1 Pyramidal Neurons

#### 2.4.1. Tumor Necrosis Factor α (TNF- α) Immunoreactivity

**Figure 4:**
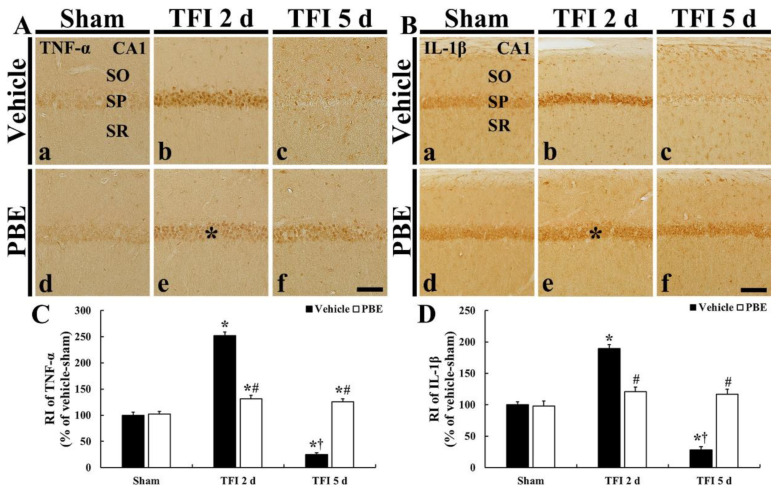
(**A**,**B**) Representative images of TNF-α (**A**) and IL-1β (**B**) immunohistochemistry in CA1 of the vehicle + sham (**a**), vehicle + TFI (**b**,**c**), PBE + sham (**d**), and PBE + TFI (**e**,**f**) groups. In the vehicle + TFI group, both TNF-α and IL-1β immunoreactivities are dramatically increased in pyramidal cells (asterisk in (**b**)) at 2 days post-ischemia and hardly shown (arrow in (**c**) at 5 days post-ischemia. However, in the PBE + TFI group, at 2 days post-ischemia, TNF-α and IL-1β immunoreactivity in the pyramidal cells (asterisks in (**e**)) is lower than that shown in the vehicle + TFI group. Scale bar = 50 μm. (**C**,**D**) RI of TNF-α (**C**) and IL-1β (**D**) immunoreactivity in CA1 pyramidal cells. The bars indicate the means ± SEM (*n* = 7 at each time; * *p* < 0.05 versus vehicle + sham group, **^#^** *p* < 0.05 versus corresponding vehicle + TIF group, and **^†^** *p* < 0.05 versus pre-time point group).

In the vehicle + sham group, weak TNF-α immunoreactivity was detected in CA1 pyramidal neurons (Figure 4Aa). In the vehicle + TFI group, TNF-α immunoreactivity in the CA1 pyramidal cells was dramatically increased (252.0% of the sham group) in the pyramidal cells at 2 days post-ischemia (Figure 4Ab,C), and, at 5 days post-ischemia, TNF-α immunoreactivity in the CA1 pyramidal cells was hardly shown because the pyramidal cells were dead at this time: in these pyramidal cells, relative immunoreactivity (RI) was 25.1% of the sham group (Figure 4Ac,C).

In the PBE + sham group, TNF-α immunoreactivity in CA1 pyramidal cells was similar to that in the vehicle + sham group (Figure 4Ad,C). In the PBE + TFI group, TNF-α immunoreactivity in CA1 pyramidal cells was slightly increased after TFI: the RI was 131.3% at 2 days and 126.0% at 5 days post-ischemia compared with that the sham group (Figure 4A(e,f),C).

#### 2.4.2. Interleukin 1β (IL-1β) Immunoreactivity

In the vehicle + sham group, IL-1β immunoreactivity was found in CA1 pyramidal cells (Figure 4Ba). In the vehicle + TFI group, IL-1β immunoreactivity in the pyramidal cells was dramatically increased (189.3% of the sham group) at 2 days post-ischemia (Figure 4Bb,D), but, at 5 days post-ischemia, IL-1β immunoreactivity in the pyramidal cells was hardly shown (27.9% of the sham group) due to their death (Figure 4Bc,D).

In the PBE + sham group, IL-1β immunoreactivity found in CA1 pyramidal cells was not different from that shown in the vehicle + sham group (Figure 4Bd,D). In the PBE + TFI group, IL-1β immunoreactivity in the pyramidal cells was slightly increased (120.8% at 2 days and 117.1% at 5 days post-ischemia compared with that in the vehicle + sham group) after TFI (Figure 4B(e,f),D).

### 2.5. PBE Increased Anti-Inflammatory Cytokines in Ischemic CA1 Pyramidal Neurons

#### 2.5.1. IL-4 Immunoreactivity

**Figure 5:**
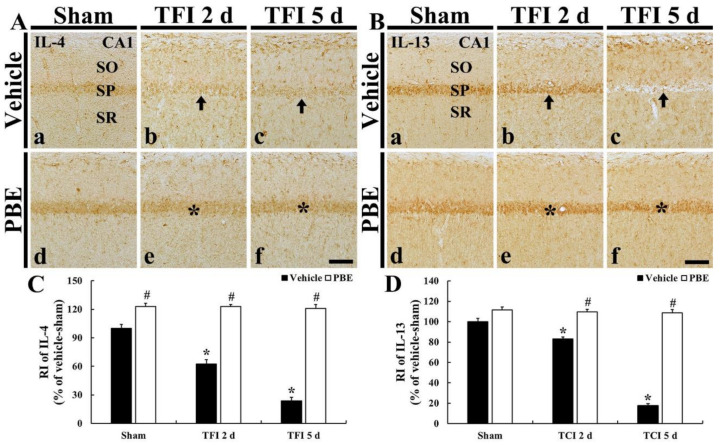
(**A**,**B**) Representative images of IL-4 (**A**) and IL-13 (**B**) immunohistochemistry in CA1 of the vehicle + sham (**a**), vehicle + TFI (**b**,**c**), PBE + sham (**d**), and PBE + TFI (**e**,**f**) groups. In the vehicle + TFI group, IL-4 and IL-13 immunoreactivity in CA1 pyramidal cells is significantly decreased (arrow in (**Ab**,**Ac**)) at 2 days and 5 days after ischemia. In the PBE + sham group, IL-4 and IL-13 immunoreactivity in CA1 pyramidal cells is slightly higher than that in the vehicle + sham group. In the PBE + TFI, increased IL-4 and IL-13 immunoreactivity in the pyramidal cells is maintained (asterisk in (**e**,**f**) after TIF. Scale bar = 50 μm. (**C**,**D**) RI of IL-4 (**C**) and IL-13 (**D**) immunoreactivity in CA1 pyramidal cells. The bars indicate the means ± SEM (*n* = 7 at each time; * *p* < 0.05 versus vehicle + sham group, **^#^** *p* < 0.05 versus corresponding vehicle + TFI group).

In the vehicle + sham group, IL-4 immunoreactivity was weakly shown in CA1 pyramidal cells (Figure 5Aa). However, in the vehicle + TFI group, IL-4 immunoreactivity in the CA1 pyramidal cells was significantly decreased (62.7% at 2 days and 23.8% at 5 days after ischemia versus the vehicle + sham group) after TFI (Figure 5A(b,c),C).

In the PBE + sham group, IL-4 immunoreactivity in CA1 pyramidal cells was significantly higher (123.1%) than that found in the vehicle + sham group (Figure 5Ad,C). In the PBE + TFI group, increased IL-4 immunoreactivity in the CA1 pyramidal cells was maintained (123.0% at 2 days and 121.0% at 5 days after ischemia versus the sham group) after TFI (Figure 5A(e,f),C).

#### 2.5.2. IL-13 Immunoreactivity

In the vehicle + sham group, IL-13 immunoreactivity was found in CA1 pyramidal cells (Figure 5Ba). In the vehicle + TFI group, IL-13 immunoreactivity in the pyramidal cells was significantly reduced at 2 days (83.1% versus the vehicle + sham group) and more decreased (17.6% versus the vehicle + sham group) at 5 days post-ischemia (Figure 5B(b,c,)D).

In the PBE + sham group, IL-13 immunoreactivity in CA1 pyramidal cells was slightly higher (111.5%) than that shown in the vehicle + sham group (Figure 5Bd,D). In the PBE + TFI group, increased IL-4 immunoreactivity in the pyramidal cells was not significantly altered after TFI compared with that in the PBE + sham group (Figure 5B(e,f,)D).

## 3. Discussion

Extracts from natural sources contain multiple biological-active compounds and have gained increasing attention due to their diverse pharmacological actions [17,18,19]. For this reason, they have been developed as commercial products for improving human health [20]. Additionally, many studies have shown the protective potential of natural products in the experimental models of neurological diseases, including brain ischemia [8,21,22].

It has been reported that pine bark extract exerts strong neuroprotective effects. For some instances, Pycnogenol^®^, which is a standardized pine bark extract originating from French maritime, shows an excellent neuroprotective effect against brain ischemic injury induced by TFI in gerbils by strong antioxidant efficacy [13]. Furthermore, the neuroprotective potential of Pycnogenol^®^ has been multifariously studied in a series of experiments in vitro and in vivo. In detail, Kobayashi et al. (2000) demonstrated that Pycnogenol^®^ protected HT4 neuronal cells (rat hippocampal cell line) from cytotoxicity induced by glutamate [23]. Furthermore, a recent study by Ozoner et al. (2019) showed that Pycnogenol^®^ alleviated the death of cortical neurons following transient focal cerebral ischemia induced by middle cerebral artery occlusion in rats [24]. Additionally, PineXol^®^, which is derived from Korean red pine bark and used as a commercialized additive for functional foods and cosmetics, protected neuronal PC-12 cells from H_2_O_2_-induced oxidative cell death by antioxidant efficacy [14].

A precedent study showed that Pycnogenol^®^ contained 70% ± 5% of standardized procyanidins [13]. Compared with this, in the present study, we quantitatively analyzed the PBE and found that the PBE contained an abundant amount of phenol, flavonoid, and proanthocyanidin. With this PBE, we examined the neuroprotective efficacy of pre-treated PBE in gerbil hippocampal CA1 after 5-min TFI using H&E staining, NeuN immunofluorescence, and F-J B histofluorescence and found that pre-treatment with 100 mg/kg, not 25 or 50 mg/kg, of PBE, effectively protected CA1 pyramidal neurons from ischemic injury. In light of the above-mentioned and our present findings, it is strongly suggested that pre-treatment with 100 mg/kg PBE protects brain neurons from transient ischemic injury. Hippocampal neuronal death in gerbils is easily induced by 5-min TFI. The hippocampus is a substructure of the brain and is deeply involved with cognitive and memory functions. In this regard, precedent studies on neuroprotective effects in a gerbil model of transient ischemia have demonstrated the amelioration of cognitive decline following TFI [13,25].

It is well addressed that when the CNS is under pathological conditions, reactive gliosis (astrogliosis and microgliosis) occurs, showing that the glial cells are proliferated and hypertrophied with thickened processes [26,27,28]. With a focus on the microglia, they act as an immunocyte in the CNS and are classified as M1 and M2 microglia [29,30]. Between the two types, M1 microglia secrete pro-inflammatory cytokines, which lead to an advance in the inflammatory response [26,30,31]. For example, some previous studies demonstrated that, under microgliosis, M1 microglia were predominantly distributed in ischemic brain regions [26,30]. In this regard, numerous researchers have searched for neuroprotective materials that considerably attenuate reactive microgliosis in the CNS following ischemic insults [17,26,32,33]. Further, our current study showed that pre-treatment with PBE significantly ameliorated TFI-induced reactive microgliosis in ischemic gerbil hippocampal CA1.

Our current findings showed that pre-treatment with 100 mg/kg PBE significantly reduced pro-inflammatory cytokines (TNF-α and IL-1β) expression in CA1 after TFI. In addition, pre-treatment with PBE significantly inhibited the decrease of anti-inflammatory cytokines (IL-4 and IL-13). It has been reported that ischemia-induced neuroinflammatory response can lead to beneficial and/or deleterious conditions in the CNS, and this is modulated by pro- and anti-inflammatory cytokines [34,35]. Namely, in ischemic CNS, pro-inflammatory cytokines exacerbate inflammatory response; whereas, anti-inflammatory cytokines generate advantageous inflammation via inhibiting the expressions of pro-inflammatory cytokines [36,37]. In this regard, accumulating experimental data have coherently shown that neuroprotective materials up-regulate anti-inflammatory cytokines and suppress pro-inflammatory cytokines in ischemic brain regions following ischemic insults. For example, it was reported that chlorogenic acid, a phenolic compound composed of ester bond between caffeic acid and quinic acid, protected pyramidal neurons in the hippocampus from ischemic injury induced by transient ischemia in gerbils by regulating such pro- and anti-inflammatory cytokines [25]. In addition, a recent study showed that Pycnogenol^®^ reduced the levels of pro-inflammatory cytokines (TNF-α and IL-1β) in the ischemic cerebral cortex induced by bilateral common carotid artery occlusion in rats [24]. Taken together, our present results correspond with the results from such precedent studies showing that pre-treatment with neuroprotective extracts or materials suppressed TFI-induced elevation of pro-inflammatory cytokines and enhanced anti-inflammatory cytokines in ischemic brains.

## 4. Materials and Methods

### 4.1. Preparation of PBE Administration

*Pinus densiflora* inhabiting Gangneung (Gangwon, Korea) maritime was cultivated, and their bark was harvested. The bark was washed with pure water and fully dried. Thereafter, using an IKA M20 grinder (IKA, Staufen, Germany), the dehydrated bark was pulverized into a fine powder. The powder was extracted with pure water (100 °C) for 24 h three times, and the extract was filtered using a filter paper (Whatman No. 1; Whatman Ltd., Maidstone, Kent, UK). Next, the extract was concentrated by a vacuum evaporator (N-12, Eyela Singapore Pte. Ltd., Singapore). The evaporated extract was rapidly freeze-dried at −55 °C using a lyophilizer (FD8512, ilShin BioBase Co. Ltd., Seoul, Korea) and stored at −20 °C.

### 4.2. Qualitative Analysis of PBE

#### 4.2.1. Total Phenol Content

Total phenol content was determined using Folin–Ciocalteu’s colorimetric method [38]. Ten percent of Folin–Ciocalteu reagent (1 mL) and sample (1 mL) were mixed. Then 1 mL of 2% sodium carbonate (Na_2_CO_3_) reagent was added, followed by mixing and incubating for 1 h in a darkroom. The absorbance was evaluated at 750 nm using a microplate reader. The total phenol content was determined from the standard curve (prepared using gallic acid).

#### 4.2.2. Total Flavonoid Content

Total flavonoid content was determined with the aluminum nitrate method [39]. To the sample (0.5 mL), 1.5 mL of 95% ethyl alcohol, 0.1 mL of 1M potassium acetate, 0.1 mL of 10% aluminum nitrate, and 2.8 mL of distilled water were added. The reaction was carried out at room temperature for 30 min, and the absorbance was evaluated at 415 nm using a microplate reader. The flavonoid content was determined from the standard curve (prepared using quercetin).

#### 4.2.3. Total Proanthocyanidin Content

Total proanthocyanidin content was measured by modifying the vanillin-hydrochloric acid method [40]. Methanol solution (l mL) containing 0.8 mg of sample was placed in a brown tube, and 6 mL of the methanol solution containing 4% vanillin was added to this. After stirring, concentrated hydrochloric acid (3 mL) was added to the solution. Thereafter, the solution was shaken every 5 min for 15 min, and the absorbance was evaluated at 490 nm using a microplate reader. The proanthocyanidin content was determined from the standard curve (prepared using catechin).

### 4.3. Experimental Animals

Six-month-old male adult gerbils weighing 72–78 g were supplied by the Experimental Animals Center (Kangwon National University, Chuncheon, Korea). The protocol for this research was approved (approval no. KW-200113-1; 18 February 2020) by Institutional Animal Care and Use Committee (IACUC) in Kangwon National University (Chuncheon, Korea). This protocol adhered to the guidelines contained in the “Current International Laws and Policies”, a part of the “Guide for the Care and Use of Laboratory Animals” of The National Academies Press (8th Ed., 2011). We did our best to minimize the total number of gerbils used in this study and to reduce their pain.

### 4.4. Experimental Groups and PBE Administration

A total of 70 gerbils were blindly and randomly divided into eight groups as follows: (1) vehicle plus (+) sham group (*n* = 7), which was treated with vehicle (sterilized normal saline; 0.85% NaCl *w*/*v*) and not subjected to TFI; (2) vehicle + TFI group (*n* = 14), which was treated with vehicle and given TFI; (3)–(5) 25, 50, and 100 mg/kg PBE + sham group (*n* = 7 respectively), which were treated with 25, 50, and 100 mg/kg of PBE, respectively, and not subjected to TFI and; (6)–(8) 25, 50, and 100 mg/kg PBE + TFI group (*n* = 7, 7, and 14, respectively), which were treated with 25, 50, and 100 mg/kg of PBE, respectively, and given TFI. PBE (25, 50, and 100 mg/kg in saline, respectively) or vehicle was orally administrated once a day for seven days before TFI induction. In this experiment, the behavior of the gerbils treated with PBE was not different from that shown in normal gerbils.

### 4.5. Induction of TFI

As previously described [41], 5 min of TFI was induced by occlusion of both common carotid arteries. In brief, the gerbils in all groups were anesthetized using a mixture of 2–2.5% isoflurane (Hana Pharm. Co., Hwaseong, Korea) in 33% oxygen and 67% nitrous oxide using an inhaler [42]. Under anesthesia, a middle incision on the neck was made, and both arteries were isolated and clamped with aneurysm clips for 5 min. To confirm perfect ischemia, the interruption of blood flow was observed in central arteries of both retinae, which are branches of the internal carotid arteries, using an ophthalmoscope (HEINE K180) (Heine Optotechnik, Herrsching, Germany). After this confirmation, the clips were removed for reperfusion. For body temperature during this operation, normal rectal temperature was controlled (37 ± 0.2 °C) using a thermometric blanket. The monitoring of temperature was done using a rectal temperature probe (TR-100) of Fine Science Tools (Foster City, CA, USA). The gerbils of the sham groups were given the surgical procedure without the occlusion of the arteries

### 4.6. Preparation of Brain Tissue Sections

As described in our published paper [43], the preparation of gerbil brain sections containing the hippocampus was carried out. Briefly, gerbils (*n* = 7 each time point after TFI) received deep anesthesia by intraperitoneal injection of 200 mg/kg pentobarbital sodium (JW pharm. Co., Ltd., Seoul, Korea) [42]. Under the anesthesia, their brains were washed by perfusion of 0.1 M phosphate-buffered saline (PBS, pH 7.4) via the ascending aorta and immediately fixed the brains with 4% paraformaldehyde (in 0.1 M PB, pH 7.4). The fixed brains were removed and stored in the same fixative. Six hours later, the brains were soaked in 30% sucrose solution for 10 h to prevent the brains from freezing injury. The cryoprotected brains were cut into frontal planes of 30 μm thickness in a cryostat. For this experiment, the sections selected were between −1.4 and −2.2 mm levels based on Bregma with reference to the gerbil brain atlas [44].

In this experiment, we had toxicological observations. The conditions of some organs (i.e., liver) of the gerbils treated with PBE were not different from those of normal gerbils.

### 4.7. H&E Staining

To examine histopathology in CA1 of all groups, H&E staining was done in the sections at 5 days after TFI; at this time, pyramidal neurons in CA1 were dead after 5-min TFI. In brief, according to a published procedure [43], the sections were put onto microscopy slides (coated with gelatin). Thereafter, these sections were stained with H&E, dehydrated with serial ethanol, and cleared with xylene. Finally, they were mounted with Canada balsam (Kanto Chemical, Tokyo, Japan).

These prepared slides were examined using a light microscope of BX53 (Olympus, Tokyo, Japan) equipped with a digital camera of DP72 (Olympus, Tokyo, Japan) connected to a PC monitor.

### 4.8. NeuN Immunofluorescence and F-J B Histofluorescence Staining

To assess the neuroprotective effects of PBE in CA1 at 5 days after TFI, neurons were stained with NeuN (a marker for neurons) by immunofluorescence staining and F-J B (a fluorescent marker for cellular damage or degeneration) by histofluorescence staining.

In brief, as described previously [41], for NeuN immunofluorescence staining, the sections were briefly rinsed and reacted with a diluted mouse anti-NeuN (1:1,000) (Chemicon, Temecula, CA, USA) at room temperature for 7 h. They were washed and reacted with a diluted Cy3-conjugated donkey anti-mouse immunoglobulin G (IgG) (1:500) (Vector Laboratories Inc., Burlingame, CA, USA) for 2.5 h at room temperature.

For F-J B histofluorescence, the sections were briefly immersed in 1% sodium hydroxide solution and incubated in 0.06% potassium permanganate solution. Thereafter, they were reacted with 0004% F-J B solution (Histochem, Jefferson, AR, USA). Finally, they were briefly washed and put onto slide warmers (50 ± 0.5 °C) for the reaction of F-J B.

NeuN-immunostained neurons and F-J B-positive cells were evaluated according to a published method [45]. Briefly, digital images of both cells were captured using a fluorescence microscope of BX53 (Olympus, Tokyo, Japan) with green and blue excitation lights (510–560 nm and 450–490 nm of wavelength, respectively). The captured neurons were counted in 250 μm^2^ at the center in CA1. Cell count was carried out by averaging the total numbers using the image analyzing system (software: Optimas 6.5) of CyberMetrics (Scottsdale, AZ, USA).

### 4.9. Immunohistochemistry

Immunohistochemical staining was performed to examine neuroinflammation using Iba-1 for microgliosis, IL-1β and TNF- α for pro-inflammatory response, and IL-4 and IL-13 for anti-inflammatory response, respectively. The sections were immunohistochemically stained according to a published protocol [46]. In short, the prepared sections were incubated with each primary antibody: rabbit anti-Iba-1 (1:900, Wako, Osaka, Japan), rabbit anti-IL-1β (1:250, Santa Cruz Biotechnology, Santa Cruz, CA, USA), rabbit anti-TNF-α (1:1200, Abcam, Cambridge, UK), rabbit anti-IL-4 (1:300, Santa Cruz Biotechnology, Santa Cruz, CA, USA), and rabbit anti-IL-13 (1:300, Santa Cruz Biotechnology, Santa Cruz, CA, USA). After washing briefly, they were reacted with biotinylated donkey anti-rabbit IgG (1:250, Vector Laboratories Inc., Burlingame, CA, USA) and horse anti-mouse IgG (1:250, Vector Laboratories Inc.) and exposed to Elite avidin-biotin enzyme complex (ABC; Vector Laboratories Inc.). Finally, these immunoreacted sections were visualized with a solution of 3,3′-diaminobenzidine tetrahydrochloride (DAB) (Sigma-Aldrich, St. Louis, MO, USA).

Negative control tests were performed to establish the specificity of each immunostaining, with preimmune serum in place of each primary antibody. As a result, any immunostained structures were not shown in the sections, although the data were not shown at present.

To quantitatively analyze the density of Iba-1, TNF-α, IL-1β, IL-4, and IL-13 immunoreactive structures, RI or ROD was used for the analysis using Adobe Photoshop (version 8.0) and NIH Image (1.59 software), as previously described by [45]. Briefly, a digital image of each immunoreactive structure was captured using the above-mentioned method. The captured image was calibrated into an array of 512 × 512 pixels. RI or ROD was evaluated on the basis of optical immunoreactivity (OI) or optical density (OD), respectively. Iba-1 immunoreactive structure was expressed as ROD, and TNF-α, IL-1β, IL-4, and IL-13 immunoreactive structures were expressed as RI.

### 4.10. Statistical Analysis

Data obtained in this experiment are expressed as the means ± standard error of the mean (SEM). All of the statistical analyses were performed with GraphPad Prism (version 5.0) of GraphPad Software (La Jolla, CA). Differences of the means among all of the groups were analyzed by two-way analysis of variance (ANOVA) with a post hoc Bonferroni’s multiple comparison tests to elucidate TFI-mediated differences among all of the groups. *p* < 0.05 was used for statistical significance.

## 5. Conclusions

In this experiment using gerbils, pre-treatment with 100 mg/kg of PBE exerts a remarkable neuroprotective effect through attenuation of TFI-induced neuroinflammatory responses (attenuation of microglia activation, inhibition of pro-inflammatory cytokine expression, and increase of anti-inflammatory cytokine expression) in hippocampal CA1 after TFI. Based on the above-mentioned previous and current results, the anti-inflammatory effect of PBE pre-treatment may contribute to protecting neurons from TFI. Therefore, PBE may be utilized as a preventive or alternative medicine for the prevention of brain ischemic damage. However, further investigation is necessary to uncover other mechanisms of PBE against ischemic brain injury.

## Figures and Tables

**Table 1:** Total polyphenols, flavonoids, and proanthocyanidins of PBE.

Total Polyphenols (mg GAE/g)	Total Flavonoids (mg QE/g)	Total Proanthocyanidins (mg CE/g)
92.89 ± 0.66	23.57 ± 0.11	53.42 ± 6.74

All values are presented by mean ± SEM. GAE, gallic acid equivalent; QE, quercetin equivalent; CE; catechin equivalent.

## Data Availability

All data produced and analyzed in the current study are included in this paper.

## Sample Availability Statement

Samples of the compounds are not available from the authors.

## Abbreviations

CA	cornu ammonis
F-J B	fluoro-Jade B
H&E	hematoxylin and eosin
IL	interleukin
NeuN	neuron-specific soluble nuclear antigen
PBE	*Pinus densiflora* bark extract
RI	relative immunoreactivity
ROD	relative optical density
SO	stratum oriens
SP	stratum pyramidale
SR	stratum radiatum
TFI	transient forebrain ischemia
TNF-α	tumor necrosis factor α
